# Photosynthetic glyceraldehyde 3‐phosphate dehydrogenase B is present in the last common ancestor of Viridiplantae predating the Streptophyta–Chlorophyta split

**DOI:** 10.1111/nph.71396

**Published:** 2026-07-21

**Authors:** Alex P. Mann, James E. M. Stach, Maxim V. Kapralov, Patricia E. Lopez‐Calcagno

**Affiliations:** ^1^ School of Natural and Environmental Sciences Newcastle University Newcastle upon Tyne NE1 7RU UK

**Keywords:** Calvin–Benson–Bassham cycle, Chlorophyta, CP12, glyceraldehyde‐3‐phosphate dehydrogenase, Ostreococcus, phylogenetics, Streptophyta, Viridiplantae

## Abstract

Predicted evolutionary history of GAPA (blue squares), CP12 (red circles), and GAPB (represented as a fusion of a blue square and half red circle) across the Viridiplantae.

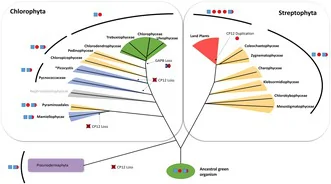

## Disclaimer

The New Phytologist Foundation remains neutral with regard to jurisdictional claims in maps and in any institutional affiliations.

## Introduction

Glyceraldehyde 3‐phosphate dehydrogenase (GAPDH) enzymes are ubiquitous across nearly all of the tree of life and have traditionally been considered ‘housekeeping genes’, frequently used as positive controls (Zhang *et al*., 2015). This enzyme is responsible for catalysing the reversible conversion of glyceraldehyde‐3‐phosphate (G3P) to 1,3‐bisphosphoglycerate using NAD(P)+, with the forward reaction occurring in glycolysis and the reverse in photosynthesis (Suzuki *et al*., 2020). In Viridiplantae, GAPDH can be grouped according to the subcellular location and function: GAPA and GAPB are plastidic NAD(P)H‐dependent enzymes which convert 1,3‐bisphosphoglycerate to G3P in the Calvin–Benson–Bassham (CBB) cycle, whereas GAPC is a cytosolic glycolytic enzyme (Zeng *et al*., 2016). An additional glycolytic GAPDH, GAPCp, is unique to land plants and is present in nonphotosynthetic plastids (Petersen *et al*., 2003).

**Fig. 1:**
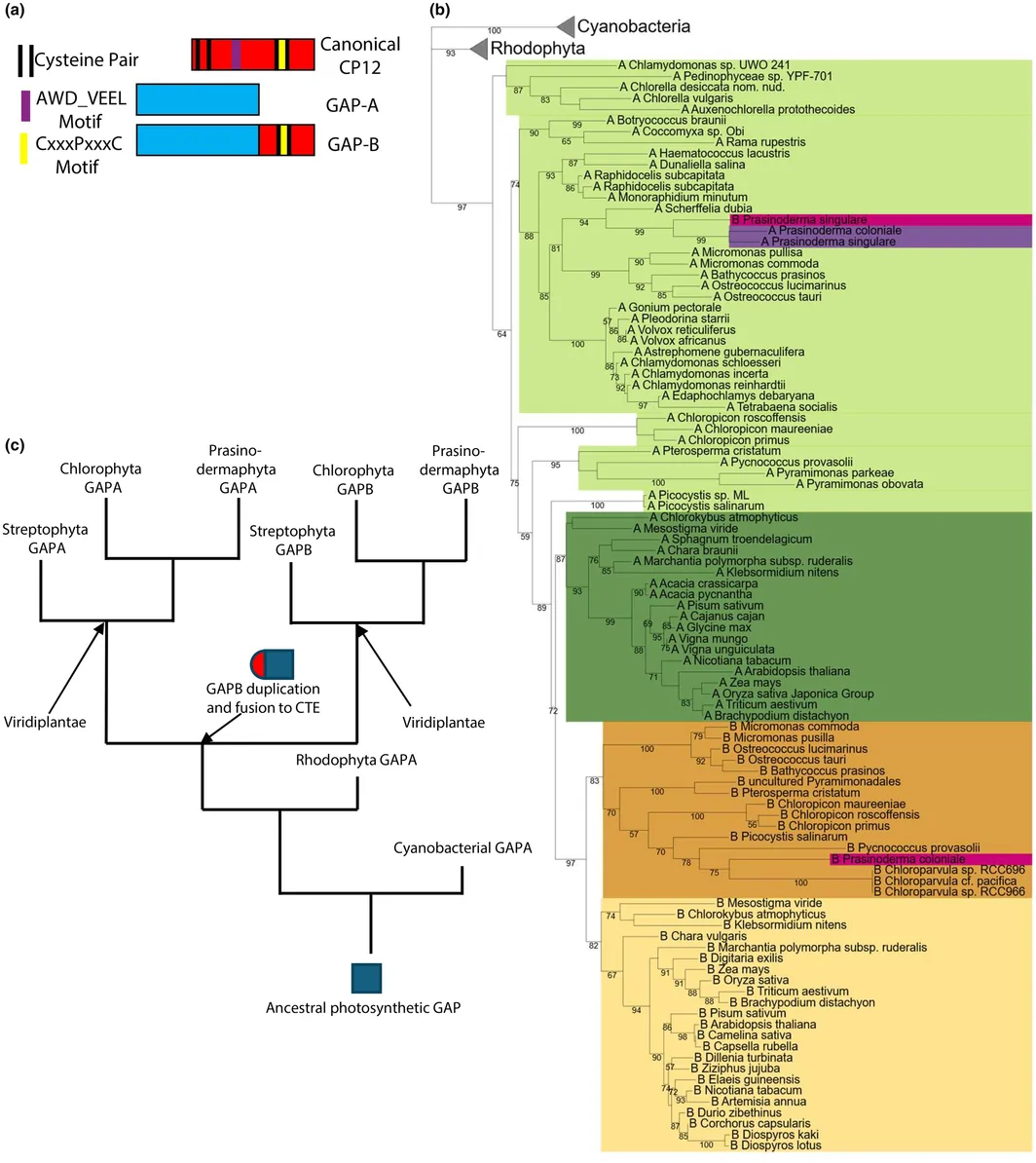
GAPB evolved from a GAPA‐CP12 fusion, and its retention varies across lineages. (a) Graphical representation of the C‐terminal extension (CTE) fusion between GAPA and CP12 to form GAPB. Red, CP12; blue, GAPA. (b) Maximum likelihood phylogeny of GAPDH proteins rooted with cyanobacterial sequences as the outgroup. The tree was constructed using an LG + G + I + F substitution model. Node support values represent Shimodaira–Hasegawa‐like approximate likelihood ratio test (SH‐aLRT) values (Anisimova & Gascuel, 2006). Colour coding: light green indicates Chlorophyta GAPA, dark green indicates Streptophyta GAPA, orange indicates Chlorophyta GAPB, and yellow indicates Streptophyta GAPB. *Prasinoderma* sequences are highlighted separately (pink for GAPB and purple for GAPA). (c) Proposed evolutionary history of GAP proteins, including the duplication event that gave rise to GAPB. This is schematic, not to scale, and does not represent chronological evolutionary time. GAPA is shown as a blue square, GAPB as a blue square with a red semicircle, and CP12 as a red circle. CP12 is present in all lineages shown.

Photosynthetic GAPDH forms tetramers of either A_4_ or A_2_B_2_ (Ferri *et al*., 1990). Unlike other GAPDHs, these enzymes preferentially use NADPH and are subject to redox regulation (Sparla *et al*., 2004). GAPDH regulation is mediated either by CP12, which links GAPDH to PRK in an inhibitory ternary complex: GAPDH_2_‐CP12_4_‐PRK_2_, where GAPDHs are tetramers and PRKs are dimers (Wedel *et al*., 1997; Wedel & Soll, 1998; Howard *et al*., 2008; Marri *et al*., 2009; Lopez‐Calcagno *et al*., 2014), or by the B subunit itself, which carries a C‐terminal extension (CTE) homologous to CP12. CP12 is an conditionally disordered protein with three key motifs: an N‐terminal cystine pair, a central AWDEEL motif responsible for PRK binding, and GAPDH binding motif CxxxPxxxxC (Trost *et al*., 2006; Lopez‐Calcagno *et al*., 2014). GAPB is thought to have arisen via duplication of GAPA and fusion with the CTE, enabling autonomous redox regulation (Fig. 1a; Ferri *et al*., 1990; Zapponi *et al*., 1993; Sparla *et al*., 2002). The CTE from GAPB mimics the scaffolding role of CP12 and lies within a cleft between GAPA and GAPB active sites, forming an inhibitory octameric (A_4_B_4_) or hexadecameric (A_8_B_8_) complexes (Fermani *et al*., 2007; Marotta *et al*., 2022). The CTE also exists in other proteins, such as ADK3, which binds GAPDH and inhibits NAPDH‐dependent activity similarly to CP12 (Thieulin‐Pardo *et al*., 2016; Zhang *et al*., 2018).

It was previously believed that GAPB was restricted to streptophytes, with the exception of the green alga *Ostreococcus*, explained by the acquisition of GAPB by horizontal gene transfer (HGT; Robbens *et al*., 2007). This positioned GAPB as a molecular marker of streptophyte origin, or alternatively necessitated an earlier origin of GAPB (Petersen *et al*., 2006; Robbens *et al*., 2007). GAPB is completely absent in most sequenced chlorophytes, with the only exception being Ostreococcus, suggesting that these species rely solely on CP12 for regulation (Robbens *et al*., 2007). By contrast, Streptophyta require dual regulation via two partially redundant but distinct mechanisms, in which GAPDH activity is redox regulated by CP12 and GAPB (Howard *et al*., 2011a,b; López‐Calcagno *et al*., 2017; Li *et al*., 2018; Simkin *et al*., 2023). GAPB independently has no interaction with PRK, and only CP12 can provide the synergy between PRK and GAPDH regulation via the ternary complex (Trost *et al*., 2006).

Here, we present new GAPDH sequences, particularly GAPBs, from a wide range of Chlorophyta as well as Prasinodermphyta species, including several early‐diverging lineages where GAPB was previously thought to be absent. Notably, Prasinodermophyta are regarded as a sister clade to Chlorophyta, diverging after the separation from Streptophyta, and therefore serve as an informative evolutionary marker within the green plant lineage (Yang *et al*., 2023). The discovery of GAPB across diverse chlorophyte taxa rules out the earlier hypothesis of HGT and instead supports the conclusion that GAPB origin predates the Streptophyta–Chlorophyta split.

### Identification of GAPBs

In this study, putative GAPA and GAPB homologues were discovered across a wide range of chlorophyte green algae where they were previously thought to be absent, including Chloropicophyceae, Pedinophyceae, Picocystis, Pycnococcaceae, and Pyramimonadales, in addition to previously established sequences from *Ostreococcus tauri* and *Ostreococcus lucimarinus* (Robbens *et al*., 2007). Additionally, GAPB sequences were found in the Prasinodermophyta (a sister clade to Chlorophyta) species: *Prasinoderma singular* and *Prasinodemra coloniale*. All sequences contained a CTE featuring the signature CxxxPxxxxC motif (Supporting Information Fig. S1). While many of the previously described GAPB‐specific marker mutations were conserved in the newly identified chlorophyte GAPBs (103G, 104P, 247N, 252G, 253T), others were not (143V); positions are referenced to Geobacillus GAPA (AAA22461.1; Fig. S2; Petersen *et al*., 2006). Another set of mutations (Fig. S3) were previously demarcated as ‘weak’ indicators of GAPB as they were conserved in only some species. The evidence from the new sequences here shows a similar pattern with few conserved residues at these points, providing limited insight into the evolutionary relationship between chlorophyte and streptophyte GAPBs (Fig. S3). The CTE remains the most reliable marker for identifying canonical GAPBs, alongside a small number of ubiquitously conserved specific mutations (Petersen *et al*., 2006).

### GAPDH evolution

Phylogenetic trees were constructed with GAPA and GAPB sequences and the observed clades are somewhat unexpected, as no single monophyletic clade encompasses all chlorophyte GAPA sequences (Fig. 1b). Instead, multiple clades are seen, which contain most chlorophyte GAPA sequences. This pattern is likely artefactual, rather than reflecting true evolutionary history and is perhaps due to the divergence of some sequences. Cyanobacteria possess a GAPA homologue (McFarlane *et al*., 2019), and it is well established that all GAPA genes in the green lineage originated from the primary endosymbiosis event. All chlorophyte, as well as streptophyte, GAPAs ultimately derive from a common ancestral gene present in the last common ancestor of the green lineage (Viridiplantae; Robbens *et al*., 2007).

GAPBs have historically been attributed to the Streptophyta lineage and are even considered a marker of Streptophyta (Petersen *et al*., 2006; Robbens *et al*., 2007). However, GAPB sequences are more common across Chlorophyta than previously expected. Rather than GAPB being restricted to a single genus of green algae or only to Streptophyta, it appears that it is only the core Chlorophyta clade which contains: Ulvophyceae, Chlorophyceae, Trebuxiophyceae, and Chlorodendrophyceae, that lacks this gene and all other Chlorophyta harbour a putative GAPB.

While Chlorophyta GAPB autoregulation is likely similar to Streptophyta GAPBs, its origin remains uncertain. There are three possible scenarios:

1. *De novo* fusion of the CTE from CP12 or another CTE‐containing domain;
2. HGT of a Streptophyta GAPB to Chlorophyta (Hunsperger *et al*., 2015; Puginier *et al*., 2024);
3. All GAPBs share a common ancestor, with canonical GAPB inherited through vertical transmission and subsequent loss in lineages that currently lack it (Wu *et al*., 2015).

### Hypothesis 1: *de novo* origin of GAPB

The *de novo* origin theory, where GAPA duplication and subsequent fusion to CTE occurs again in a separate lineage, has been ruled out previously (Robbens *et al*., 2007), and the results obtained here further support this conclusion. Chlorophyta GAPBs share conserved GAPB‐specific motifs, and the similarity between GAPB sequences across the phylogenetic trees is a strong support for the existence of a common ancestor. The *de novo* creation of GAPB must be excluded as a credible explanation, as a fusion followed by convergent evolution forming a monophyletic clade is highly improbable.

### Hypothesis 2: HGT of GAPB

The HGT of GAPB from Streptophyta to Chlorophyta (e.g. *Ostreococcus*) has historically been the established explanation for the presence of GAPB in Chlorophyta (Robbens *et al*., 2007). This remains a theoretical possibility, particularly given that all Chlorophyta GAPB sequences cluster within the same well‐supported monophyletic clade as Streptophyta GAPBs, suggesting a shared origin (Fig. 1b). However, *Ostreococcus* GAPB sequences lack typical markers of HGT, including different GC content (Fig. S4) and codon usage patterns (Table S1). Endogenous GAPBs from *Ostreococcus* did not exhibit codon usage patterns similar to those of streptophyte GAPBs, suggesting that they are not similarly codon‐optimised and are endogenous *Ostreococcus* genes. While it is possible that HGT makers were lost, there is no evidence to support recent or detectable HGT (Lawrence & Ochman, 1997; Ravenhall *et al*., 2015). Furthermore, chlorophyte GAPBs form a distinct monophyletic clade, separate from streptophyte GAPBs, which makes it unlikely that the gene originated via HGT from a streptophyte species (Fig. 1b). In the highly unlikely scenario that multiple HGT events had occurred, one would expect chlorophyte sequences to be interspersed among Streptophyta GAPBs, which is not the case (Philippe & Douady, 2003). Furthermore, as a sister clade to Chlorophyta, Prasinodermophyta are the earliest diverging family from Streptophyta and hence presence of GAPB excludes HGT.

### Hypothesis 3: GAPB was present in the common ancestor of Viridiplantae and was subsequently lost in Ulvophyceae, Chlorophyceae, Trebuxiophyceae, and Chlorodendrophyceae

Excluding the alternative hypotheses, Hypothesis 3, that GAPB was present in the common ancestor of Viridiplantae, is the most likely explanation. This is strongly supported by phylogenetic analyses, which consistently show a well‐supported Chlorophyta GAPB monophyletic clade contained within a GAPB monophyletic clade, including early Prasinodermophyta GAPB sequences, a sister clade to Chlorophyta (Fig. 1b; Li *et al*., 2020; Yang *et al*., 2023). The occurrence of GAPB in Prasinodermophyta can only be logically explained by its presence in the common ancestor of the Chlorophyta and Prasinodermophyta; similarly the presence in both early‐diverging Chlorophyta and Streptophyta suggest that GAPB was present in Viridiplantae (or multiple HGT events occurred). Excluding HGT, this finding supports the idea that GAPB originated during the earliest stages of green plant evolution.

The placement of a *Prasinoderma singulare* GAPB sequence within the Chlorophyta GAPA clade is inconsistent with its sequence features (Figs 1b, S3). This sequence possesses characteristic GAPB markers, including the CTE, a threonine insertion at position 253, and Q247N and V252G substitutions relative to the GAPA of the same species. These features strongly indicate that the sequence is a GAPB homologue, suggesting that its phylogenetic placement is likely artefactual rather than reflective of its evolutionary origin. The GAPB from *Prasinoderma coloniale* is located within the Chlorophyta GAPB clade, but it is not basal as would be expected. This is also the case for GAPAs from Prasinodermophyta.

### A model for GAPA, GAPB, and CP12: appearance and loss

**Fig. 2:**
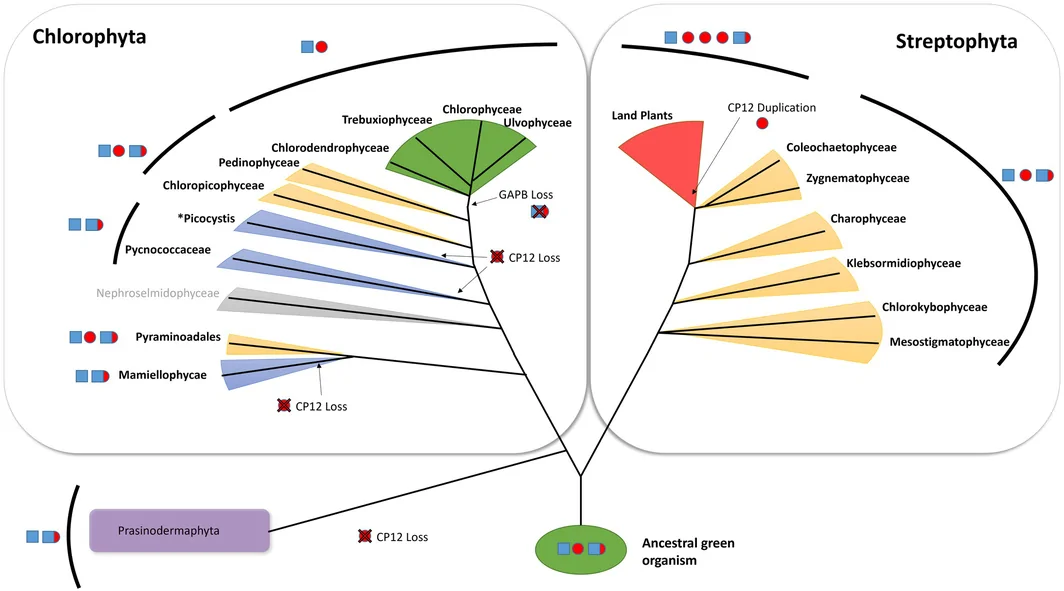
Predicted evolutionary history of GAPA (blue squares), CP12 (red circles), and GAPB (represented as a fusion of a blue square and half red circle) across the Viridiplantae. Gains and losses of CP12 and GAPB are indicated on the phylogeny. Greyed‐out family names represent lineages for which no sequence data were available. Green‐shaded families represent species without GAPB, blue‐shaded families those without CP12, yellow‐shaded families contain all three proteins, and red‐shaded families have all three proteins plus additional CP12s. Prasinodermophyta are shown as a sister clade to Chlorophyta. Picocystis is marked with an asterisk to indicate that, although it contains a canonical CP12 sequence, this sequence lacks a start codon. Relationships between taxonomic groups based on Leliaert *et al*. (2012) and Yang *et al*. (2023).

An ancestor of the entire green lineage likely contained only GAPA and CP12, similar to what is observed in cyanobacteria and red algae (Figge *et al*., 1999; Groben *et al*., 2010; Stanley *et al*., 2013; Gérard *et al*., 2022a). At some point, the GAPA gene duplicated and one copy underwent fusion with the CTE from CP12 (or other source, such as ADK3) and acquired GAPB‐specific mutations, forming what is now known as canonical GAPB (Fig. 1c). The common ancestor of the green lineage would therefore have contained canonical forms of GAPA, GAPB, and CP12 (Fig. 2).

Following the divergence of Chlorophyta and Streptophyta, both lineages initially retained all three proteins: GAPA, GAPB, and CP12. In Streptophyta, these genes have been consistently maintained, with CP12 undergoing duplication in land plants. While there is clearly some functional redundancy between CP12 and GAPB, highlighted by their similar mechanisms of regulating GAPDH, their necessity appears to vary (Trost *et al*., 2006). In land plants, both proteins seem to be essential for normal growth perhaps due to CP12's role as a moonlighting protein with multiple other functions. These moonlighting functions include altering carbon metabolism, thermotolerance, and acting as a chaperone to PRK and GAPDH, among others (Erales *et al*., 2009; Howard *et al*., 2011a,b; López‐Calcagno *et al*., 2017; Li *et al*., 2018; Simkin *et al*., 2023).

To confirm the presence or absence of CP12 in each family, databases were searched using *Chlamydomonas reinhardtii* CP12 (A6Q0K5.2) and *Arabidopsis thaliana* CP12 (O22914.1) as BLAST inputs. Most outputs returned a few hits although mostly for GAPB or other short regions of homology. Only putative CP12s with the central AWDEEL motif and both cysteine pairs were kept for analysis and included further, as listed in Fig. S5 and Table S2. Nephroselmidaceae gave no hits at all for CP12, GAPA, or GAPB, although the absence of all three remains extraordinarily unlikely. This reiterates that the absence of evidence is not evidence of absence and should any further genes or other species be sequenced the model should be revisited.

Some Chlorophyta show evidence that, beyond their functional redundancy, only one of GAPB or CP12 may be necessary (Fig. 2). For example, species from Ulvophyceae, Chlorophyceae, Trebuxiophyceae, and Chlorodendrophyceae, retain CP12 but have lost GAPB, whereas Mamiellophyceae, Pycnococcus, Prasinodermophyta, and potentially Picocystis retain GAPB but lack CP12 (Table S2; Fig. 2). In Picocystis, a potential CP12‐like sequence was identified; however, it lacked a start codon, which may indicate the early stages of CP12 loss. In Pedinophyceae which has both GAPB and CP12, there is loss of the C‐terminal GAPDH binding motif, CxxxPxxxC, in CP12 due to an additional insertion of two residues and the loss of the central proline, although the cysteine pair remains (Yu *et al*., 2020). This loss may only be possible due to the redundancy that exists between GAPB and CP12. Other CP12 sequences have maintained their GAPDH binding domain, with the PRK binding domain remaining conserved throughout Chlorophyta (Fig. S5).

Based on the new data presented here, we propose that this loss of GAPB occurred after the divergence of Pedinophyceae but before the evolution of the core Chlorophyta clade. Furthermore, experimental evidence supports the redundancy between CP12 and GAPB through their mechanism of inhibition of GAPDH tetramer. Some studies even suggest that neither are required for unhindered growth at all (under normal conditions): ΔCP12 *Chlamydomonas* mutants, which lack both CP12 and GAPB, grow similarly to wild‐type under normal conditions, suggesting that the presence of either protein is not strictly essential in all environments (Gérard *et al*., 2022b). Cyanobacteria CP12 mutants appear to grow normally under constant light but grow slower under day–night cycles, placing CP12 as the main redox regulator of the CBB cycle in cyanobacteria (Tamoi *et al*., 2005; Blanc‐Garin *et al*., 2022; Lucius *et al*., 2022).

It appears that CP12 has been lost independently in multiple lineages, suggesting that reliance on GAPB as the sole redox regulator of the CBB cycle is possible and a more frequent event than previously thought. The absence of CP12 and/or GAPB in fully sequenced lineages highlights that independent loss events are a real and significant feature of algal evolution. However, in land plants the absence of these regulatory proteins (either CP12 or GAPB) does result in severe growth phenotypes under day–night conditions, which is perhaps why plants have three redundant duplicated CP12s (López‐Calcagno *et al*., 2017; Simkin *et al*., 2023). It has been suggested that GAPB provides tighter regulation of the Calvin cycle, helping to prevent futile cycling with the oxidative pentose phosphate pathway, although CP12 alone is also capable of this. GAPB may additionally support more efficient starch mobilisation by limiting glycolytic degradation of glyceraldehyde 3‐phosphate (Petersen *et al*., 2006). In *Arabidopsis*, mutants lacking either GAPB or CP12 show reduced photosynthetic efficiency, whereas chlorophytes can tolerate the loss of one or the other in an evolutionary context; however, this has not been directly addressed by mutational studies (López‐Calcagno *et al*., 2017; Simkin *et al*., 2023). This suggests that layered regulation is required in streptophytes but may be less critical in chlorophytes. One possible explanation is that carbohydrate partitioning between tissues is more important in multicellular streptophytes, whereas in unicellular chlorophytes, redox regulation may be the dominant controlling factor.

One possible explanation for this difference lies in the sessile nature of land plants, which have limited capacity to avoid high light exposure or control light intensity reaching their leaf surface. By contrast, many green algae, especially motile forms with flagella, can escape high light through negative phototaxis and also have circadian controlled movement towards light sources for example in *Chlamydomonas* species (Mittag *et al*., 2005; Erickson *et al*., 2015). Within the Prasinophytes, most species are flagellated and capable of phototaxis (Leliaert, 2019). Most species that have lost either GAPB (e.g. core clade) or CP12, for example Mamiellophyceae and Prasinodermophyta are capable of movement (Li *et al*., 2020; Yung *et al*., 2022). However, *Chloropicon* species, which lack flagella (Lopes dos Santos *et al*., 2017) and therefore cannot perform phototaxis, have retained all three proteins. This suggests that in the absence of phototactic ability, both GAPB and CP12 may be crucial for regulating photosynthesis and responding to light stress.

Of course, this relationship is not absolute. Some Charophytes and many motile Prasinophytes, which also exhibit phototaxis, retain all three proteins (Daugbjerg *et al*., 2000; Kreimer, 2009; Domozych & Bagdan, 2022). Furthermore, *Picocystis and Pycnococcus* also lack flagella and hence the ability to move phototactically (Pálmai *et al*., 2020). This suggests that while motility and the ability to mitigate light stress through phototaxis may reduce the selective pressure to retain certain regulatory proteins, these traits alone do not determine their retention or loss.

## Conclusions

Together, the phylogenetic evidence presented in this study supports a revised model of GAPDH evolution in the green lineage. Contrary to earlier assumptions that GAPB was exclusive to Streptophyta, our findings demonstrate that canonical GAPBs, defined by a conserved CTE, are more widespread across Chlorophyta than previously recognised. The presence of GAPB in Prasinodermophyta further supports its ancient origin in the last common ancestor of both Chlorophyta and Viridiplantae. Losses of GAPB or CP12 in distinct Chlorophyta clades, alongside evidence of functional redundancy, highlight a flexible regulatory system shaped by evolutionary pressures, such as motility. This flexibility contrasts with the conserved requirement for both proteins in land plants, likely reflecting their sessile lifestyle. Our model thus provides a coherent framework for understanding the origin, diversification, and lineage‐specific retention or loss of GAPA, GAPB, and CP12 across the green lineage.

Description of Materials and Methods as well as a list of all the GAPDH genes used for the phylogenetic and sequence analyses can be found in Methods S1 and Table S3.

## Competing interests

None declared.

## Author contributions

APM designed the experiments with support from JEMS, MVK and PEL‐C. APM performed the sequence acquisition and screening and carried out the phylogenetic analyses. APM analysed the data and wrote the first draft of the manuscript. JEMS, MVK and PEL‐C revised the manuscript.

## Supporting information


**Fig. S1** Conservation and Alignment of GAPB across Chlorophyta and Streptophyta.
**Fig. S2** Mutations of GAPB‐specific residues following a MUSCLE alignment of GAPA and GAPB protein sequences.
**Fig. S3** Less conserved mutations of GAPB‐specific residues following a MUSCLE alignment of GAPA and GAPB protein sequences.
**Fig. S4** Rolling GC content across Chlorophyta chromosomes/scaffolds containing GAPB.
**Fig. S5** CP12 protein sequences from representative Chlorophyta.

**Methods S1** Supporting materials and methods.
**Table S1** Codon Adaption Index of GAPA and GAPB sequences using native and foreign codon usage tables.
**Table S2** List of key species from each family in Chlorophyta and accession numbers for their GAPA, GAPB, and CP12 sequences.
**Table S3** List of GAPDH genes used in phylogenetic and sequence analyses.
Please note: Wiley is not responsible for the content or functionality of any Supporting Information supplied by the authors. Any queries (other than missing material) should be directed to the *New Phytologist* Central Office.

## Data Availability

All data used are publicly available; references to this are present in Figs S1–S5 and Tables S1–S3.
