# Supplementary material for: Photosynthetic glyceraldehyde 3‐phosphate dehydrogenase B is present in the last common ancestor of Viridiplantae predating the Streptophyta–Chlorophyta split
Source: New Phytol. 2026 Jul 21;251(6):3072–9. doi: 10.1111/nph.71396 (PMC13491260; doi:10.1111/nph.71396)
Supplement: Supplementary file 1 [file NPH-251-3072-s001.pdf]

**New Phytologist Supporting Information**

**Article title:** Photosynthetic Glyceraldehyde 3-phosphate Dehydrogenase B is present in the last common ancestor of Viridiplantae predating the Streptophyta-Chlorophyta split

**Authors**: Alex P. Mann, James E. M. Stach, Maxim V. Kapralov, Patricia E. Lopez-Calcagno

**Article acceptance date**: 19 May 2026

The following Supporting Information is available for this article:

**Figure S1 -** Conservation and Alignment of GAPB across Chlorophyta and Streptophyta.

**Figure S2 -** Mutations of GAPB-specific residues following a MUSCLE alignment of GAPA and GAPB protein sequences.

**Figure S3 -** Less conserved mutations of GAPB-specific residues following a MUSCLE alignment of GAPA and GAPB protein sequences.

**Figure S4 -** Rolling GC content across Chlorophyta chromosomes/scaffolds containing GAPB.

**Figure S5 -** CP12 protein sequences from representative Chlorophyta.

**Table S1** - Codon Adaption Index (CAI) of GAPA and GAPB sequences using native and foreign codon usage tables.

**Table S2 -** List of key species from each family in Chlorophyta and accession numbers for their GAPA, GAPB, and CP12 sequences.

**Table S3 -** List of GAPDH genes used in phylogenetic and sequence analyses.

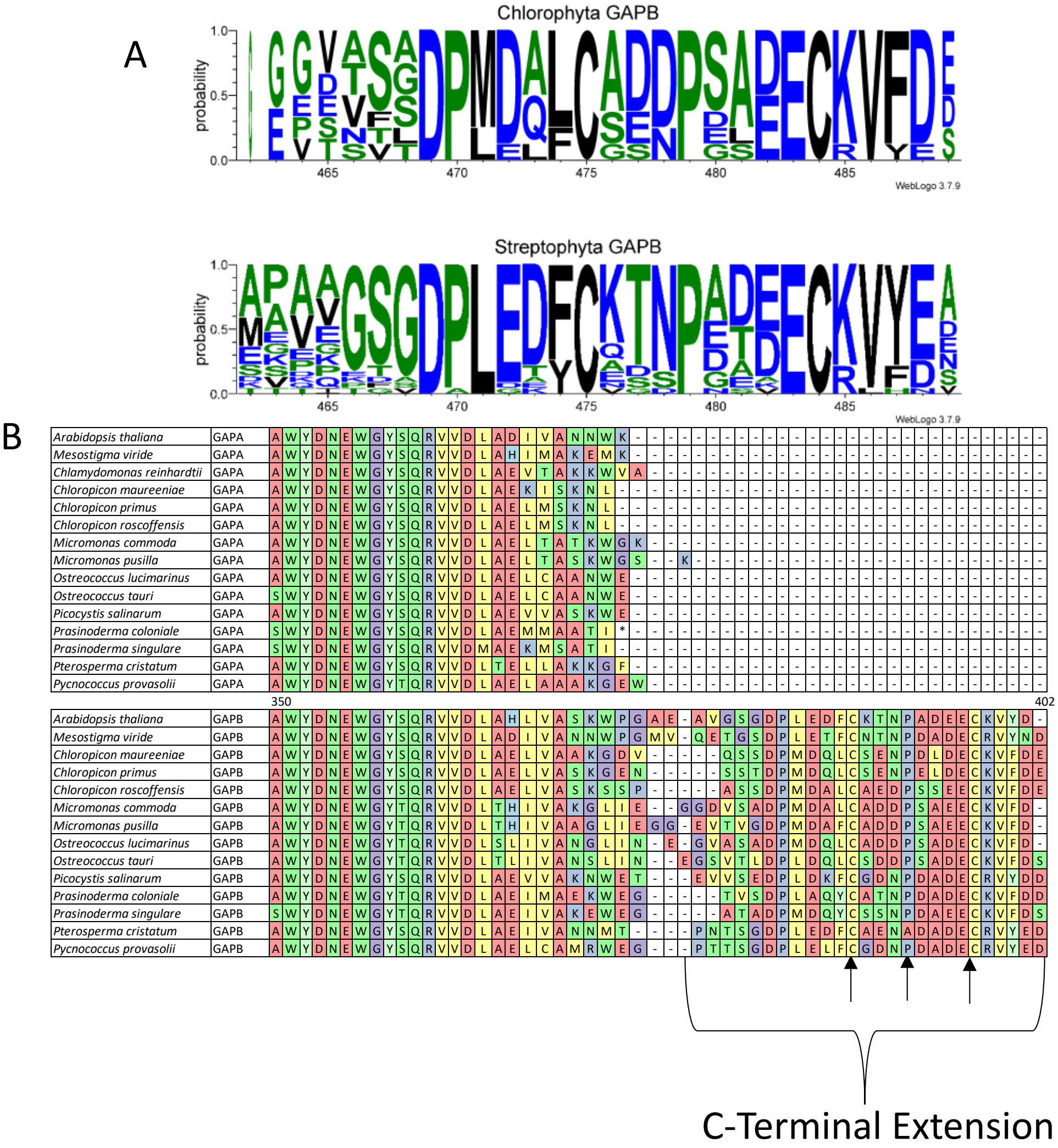


Figure S1 - Conservation and Alignment of GAPB across Chlorophyta and Streptophyta **(A)** Probability of each amino acid at each position in the C-terminal extension (CTE) region of GAPB sequences from Chlorophyta and Streptophyta. *Geobacillus* GAPDH was used to determine the reference position numbering. Hydrophobic residues are shown in black, polar or charged residues in blue and other residues in green, **(B)** MUSCLE alignment of GAPA and GAPB C-terminal regions from Streptophyta: *Arabidopsis thaliana* and *Mesostigma viride*; Chlorophyta: *Chloropicon maureeniae*, *Chloropicon primus*, *Chloropicon roscoffensis*, *Chlamydomonas reinhardtii*, *Micromonas commoda*, *Micromonas pusilla*, *Ostreococcus lucimarinus*, *Ostreococcus tauri*, *Picocystis salinarum*, *Pterosperma cristatum*, *Pycnococcus provasolii*; and *Prasinodermophyta: Prasinoderma singularis* and *Prasinoderma coloniale*, referenced to the *Arabidopsis thaliana* GAPB sequence. The CTE region and key residues within it are highlighted.

| | | | 273 | 384 | 229 | 230 | 377 | 382 |
|---|---|---|---|---|---|---|---|---|
| | | | Insertion | | Substitutions | | | |
| | | Species | 143 | 253 | 103 | 104 | 247 | 252 |
| GAPB | Chlorophyta | *Bathycoccus prasinos* | - | T | G | P | N | G |
| | | *Chloroparvula cf. pacifica RCC999* | - | T | G | P | N | G |
| | | *Chloroparvula sp. RCC696* | - | T | G | P | N | G |
| | | *Chloropicon maureeniae* | - | T | T | I | N | G |
| | | *Chloropicon primus* | - | T | T | I | N | G |
| | | *Chloropicon roscoffensis* | - | T | T | I | N | G |
| | | *Micromonas commoda* | - | T | G | P | N | G |
| | | *Micromonas pusilla* | - | T | G | P | N | G |
| | | *Ostreococcus lucimarinus* | - | T | G | P | N | G |
| | | *Ostreococcus tauri* | - | T | G | P | N | G |
| | | *Picocystis salinarum* | - | T | S | S | N | G |
| | | *Pterosperma cristatum* | - | T | G | P | N | G |
| | | *Pycnococcus provasolii* | - | S | S | K | N | G |
| | Prasinodermaphyta | *Prasinoderma coloniale* | S | T | T | P | N | G |
| | | *Prasinoderma singulare* | - | T | S | E | N | G |
| | Streptophyta | *Arabidopsis thaliana* | V | T | G | P | N | G |
| | | *Chara vulgaris* | V | T | G | P | N | G |
| | | *Chlorokybus atmophyticus* | A | T | G | P | N | G |
| | | *Klebsormidium nitens* | V | T | G | P | N | N |
| | | *Marchantia polymorpha subsp. ruderalis* | V | T | G | P | N | G |
| | | *Mesostigma viride* | V | T | G | P | N | G |
| | | *Nicotiana tabacum* | V | S | G | P | N | G |
| | | *Oryza sativa* | V | T | G | P | N | G |
| | | *Lathyrus oleraceus* | V | S | G | P | N | G |
| | | *Triticum aestivum* | V | T | G | P | N | G |
| | | *Zea mays* | V | T | G | P | N | G |
| GAPA | Chlorophyta | *Chloropicon maureeniae* | - | - | T | E | Q | T |
| | | *Chloropicon primus* | - | - | T | E | Q | T |
| | | *Chloropicon roscoffensis* | - | - | S | E | Q | T |
| | | *Micromonas commoda* | - | - | T | P | Q | C |
| | | *Micromonas pusilla* | - | - | T | P | Q | C |
| | | *Ostreococcus lucimarinus* | - | - | T | P | Q | V |
| | | *Ostreococcus tauri* | - | - | T | P | Q | C |
| | | *Picocystis salinarum* | - | - | E | A | Q | T |
| | | *Pterosperma cristatum* | - | - | E | A | Q | T |
| | | *Pycnococcus provasolii* | - | - | Q | A | Q | T |
| | Prasinodermaphyta | *Prasinoderma coloniale* | - | - | K | E | Q | V |
| | | *Prasinoderma singulare* | - | - | S | E | Q | V |
| | Chlorophyta | *Astrephomene gubernaculifera* | - | - | K | V | Q | T |
| | | *Auxenochlorella protothecoides* | - | - | R | K | Q | T |
| | | *Botryococcus braunii* | - | - | S | E | Q | T |
| | | *Chlamydomonas incerta* | - | - | K | V | Q | T |
| | | *Chlamydomonas reinhardtii* | - | - | K | V | Q | T |
| | | *Chlamydomonas schloesseri* | - | - | K | V | Q | T |
| | | *Chlamydomonas sp. UWO 241* | - | - | V | K | Q | T |
| | | *Chlorella desiccata (nom. nud.)* | - | - | T | K | Q | T |
| | | *Chlorella vulgaris* | - | - | R | P | Q | T |
| | | *Coccomyxa sp. Obi* | - | - | S | E | Q | T |
| | | *Coccomyxa sp. Obi* | - | - | T | P | Q | V |
| | | *Dunaliella salina* | - | - | T | P | Q | T |
| | | *Edaphochlamys debaryana* | - | - | K | V | Q | T |
| | | *Gonium pectorale* | - | - | K | V | Q | T |
| | | *Haematococcus lacustris* | - | - | T | P | Q | T |
| | | *Monoraphidium minutum* | - | - | T | P | Q | T |
| | | *Pleodorina starrii* | - | - | K | V | Q | T |
| | | *Rama rupestris* | - | - | E | E | Q | T |
| | | *Raphidocelis subcapitata* | - | - | T | P | Q | T |
| | | *Scherffelia dubia* | T | - | S | E | Q | T |
| | | *Tetrabaena socialis* | - | - | R | V | Q | T |
| | | *Volvox africanus* | - | - | K | V | Q | T |
| | | *Volvox reticuliferus* | - | - | K | V | Q | T |
| | Streptophyta | *Acacia crassicarpa* | - | - | R | E | Q | T |
| | | *Acacia pycnantha* | - | - | R | E | Q | T |
| | | *Arabidopsis thaliana* | - | - | R | E | Q | T |
| | | *Brachypodium distachyon* | - | - | R | D | Q | T |
| | | *Cajanus cajan* | - | - | R | E | Q | T |
| | | *Chara braunii* | - | - | R | E | Q | T |
| | | *Chlorokybus atmophyticus* | - | - | S | A | Q | T |
| | | *Glycine max* | - | - | R | E | Q | T |
| | | *Klebsormidium nitens* | - | - | E | A | Q | T |
| | | *Marchantia polymorpha subsp. ruderalis* | - | - | K | E | Q | T |
| | | *Mesostigma viride* | - | - | E | A | Q | T |
| | | *Nicotiana tabacum* | - | - | R | E | Q | T |
| | | *Oryza sativa* | - | - | R | D | Q | T |
| | | *Lathyrus oleraceus* | - | - | R | E | Q | T |
| | | *Sphagnum troendelagicum* | - | - | K | E | Q | T |
| | | *Triticum aestivum* | - | - | R | A | Q | T |
| | | *Vigna mungo* | - | - | R | E | Q | T |
| | | *Vigna unguiculata* | - | - | R | E | Q | T |
| | | *Zea mays* | - | - | R | E | Q | T |
| | Bacteria | *Geobacillus stearothermophilus* | A | - | R | E | E | V |

Figure S2 - Mutations of GAPB-specific residues following a MUSCLE alignment of GAPA and GAPB protein sequences. Gaps in the alignment are indicated by (–). Prasinodermophyta sequences are highlighted in grey, as their classification within Chlorophyta remains uncertain. Positions refer to the reference *Geobacillus* sequence. Amino acids are colour-coded according to their chemical properties: A, D, and E are shown in red (acidic); I, L, M, V, F, and C in yellow (hydrophobic); S, T, Q, W, N and Y in green (polar); H in pale blue; K, R, and P in light blue (basic or structurally unique); and G in purple.

| | | | 165 | 208 | 218 | 244 | 275 | 387 |
|---|---|---|---|---|---|---|---|---|
| | | | Proposed Substitutions | | | | | |
| | | Species | 40 | 82 | 92 | 118 | 145 | 256 |
| GAPB | Chlorophyta | *Bathycoccus prasinos* | P | K | I | V | N | E |
| | | *Chloroparvula cf. pacifica RCC999* | A | T | L | L | P | D |
| | | *Chloroparvula sp. RCC696* | A | T | L | L | P | D |
| | | *Chloropicon maureeniae* | Q | Q | M | V | P | D |
| | | *Chloropicon primus* | Q | Q | M | V | P | D |
| | | *Chloropicon roscoffensis* | Q | Q | M | V | P | D |
| | | *Micromonas commoda* | P | N | I | V | N | E |
| | | *Micromonas pusilla* | P | N | I | V | N | E |
| | | *Ostreococcus lucimarinus* | P | N | I | V | N | E |
| | | *Ostreococcus tauri* | P | N | I | V | N | E |
| | | *Picocystis salinarum* | S | T | I | V | N | D |
| | | *Pterosperma cristatum* | Q | K | I | V | N | E |
| | | *Pycnococcus provasolii* | P | Q | L | L | P | A |
| | Prasinodermaphyta | *Prasinoderma coloniale* | S | A | I | L | D | D |
| | | *Prasinoderma singulare* | Q | Q | L | L | T | D |
| | Streptophyta | *Arabidopsis thaliana* | N | K | I | I | N | D |
| | | *Chara vulgaris* | Q | K | I | I | N | D |
| | | *Chlorokybus atmophyticus* | Q | Q | L | L | A | Q |
| | | *Klebsormidium nitens* | Q | K | I | I | D | E |
| | | *Marchantia polymorpha subsp. ruderalis* | N | K | I | I | N | D |
| | | *Mesostigma viride* | Q | Q | L | L | N | E |
| | | *Nicotiana tabacum* | N | K | I | I | N | D |
| | | *Oryza sativa* | N | K | I | I | N | D |
| | | *Lathyrus oleraceus* | N | K | I | I | D | D |
| | | *Triticum aestivum* | N | K | I | I | N | D |
| | | *Zea mays* | N | K | I | I | D | D |
| GAPA | Chlorophyta | *Chloropicon maureeniae* | Q | Q | L | L | K | E |
| | | *Chloropicon primus* | Q | Q | L | L | K | E |
| | | *Chloropicon roscoffensis* | Q | Q | L | L | Q | E |
| | | *Micromonas commoda* | Q | Q | L | L | K | E |
| | | *Micromonas pusilla* | Q | Q | I | I | K | D |
| | | *Ostreococcus lucimarinus* | Q | Q | I | V | K | E |
| | | *Ostreococcus tauri* | Q | Q | L | L | K | E |
| | | *Picocystis salinarum* | Q | K | L | L | T | E |
| | | *Pterosperma cristatum* | Q | E | I | V | P | E |
| | | *Pycnococcus provasolii* | Q | E | L | L | T | E |
| | Prasinodermaphyta | *Prasinoderma coloniale* | Q | A | L | L | T | S |
| | | *Prasinoderma singulare* | Q | Q | L | L | T | S |
| | Chlorophyta | *Astrephomene gubernaculifera* | Q | Q | L | L | P | E |
| | | *Auxenochlorella protothecoides* | Q | A | L | I | D | E |
| | | *Botryococcus braunii* | Q | A | L | L | N | E |
| | | *Chlamydomonas incerta* | Q | Q | L | L | P | E |
| | | *Chlamydomonas reinhardtii* | Q | Q | L | L | P | E |
| | | *Chlamydomonas schloesseri* | Q | Q | L | L | P | E |
| | | *Chlamydomonas sp. UWO 241* | Q | Q | L | L | P | E |
| | | *Chlorella desiccata (nom. nud.)* | Q | Q | L | L | K | E |
| | | *Chlorella vulgaris* | Q | K | L | L | D | E |
| | | *Coccomyxa sp. Obi* | Q | E | L | L | P | E |
| | | *Coccomyxa sp. Obi* | Q | Q | I | V | K | E |
| | | *Dunaliella salina* | Q | Q | L | L | P | E |
| | | *Edaphochlamys debaryana* | Q | Q | L | L | P | E |
| | | *Gonium pectorale* | Q | Q | L | L | P | E |
| | | *Haematococcus lacustris* | Q | T | L | L | P | E |
| | | *Monoraphidium minutum* | Q | K | L | L | T | E |
| | | *Pleodorina starrii* | Q | A | L | L | P | E |
| | | *Rama rupestris* | Q | K | L | I | P | E |
| | | *Raphidocelis subcapitata* | Q | Q | L | L | A | E |
| | | *Scherffelia dubia* | Q | K | I | V | H | E |
| | | *Tetrabaena socialis* | Q | N | L | V | P | E |
| | | *Volvox africanus* | Q | S | L | L | P | E |
| | | *Volvox reticuliferus* | Q | A | L | L | P | E |
| | Streptophyta | *Acacia crassicarpa* | Q | N | L | L | P | E |
| | | *Acacia pycnantha* | Q | N | L | L | P | E |
| | | *Arabidopsis thaliana* | Q | L | I | I | P | E |
| | | *Brachypodium distachyon* | Q | N | L | L | T | E |
| | | *Cajanus cajan* | Q | N | L | L | P | E |
| | | *Chara braunii* | Q | N | L | L | T | E |
| | | *Chlorokybus atmophyticus* | Q | D | L | L | P | E |
| | | *Glycine max* | Q | N | L | L | P | E |
| | | *Klebsormidium nitens* | Q | K | L | L | K | E |
| | | *Marchantia polymorpha subsp. ruderalis* | Q | N | L | L | Q | E |
| | | *Mesostigma viride* | Q | S | I | L | T | E |
| | | *Nicotiana tabacum* | Q | N | L | L | P | E |
| | | *Oryza sativa* | Q | N | L | L | P | E |
| | | *Lathyrus oleraceus* | Q | N | L | L | D | E |
| | | *Sphagnum troendelagicum* | Q | N | L | L | E | E |
| | | *Triticum aestivum* | Q | N | L | L | T | E |
| | | *Vigna mungo* | Q | N | L | L | P | E |
| | | *Vigna unguiculata* | Q | N | L | L | P | E |
| | | *Zea mays* | Q | N | L | L | P | E |
| | Bacteria | *Geobacillus stearothermophilus* | T | N | I | I | H | E |

Figure S3 - Less conserved mutations of GAPB-specific residues following a MUSCLE alignment of GAPA and GAPB protein sequences. Prasinodermophyta sequences are highlighted in grey, as their classification within Chlorophyta remains uncertain. Positions refer to the reference *Geobacillus* sequence. Amino acids are colour-coded according to their chemical properties: A, D, and E are shown in red (acidic); I, L, M, V, F, and C in yellow (hydrophobic); S, T, Q, W, N, and Y in green (polar); H in pale blue; K, R, and P in light blue (basic or structurally unique); and G in purple.

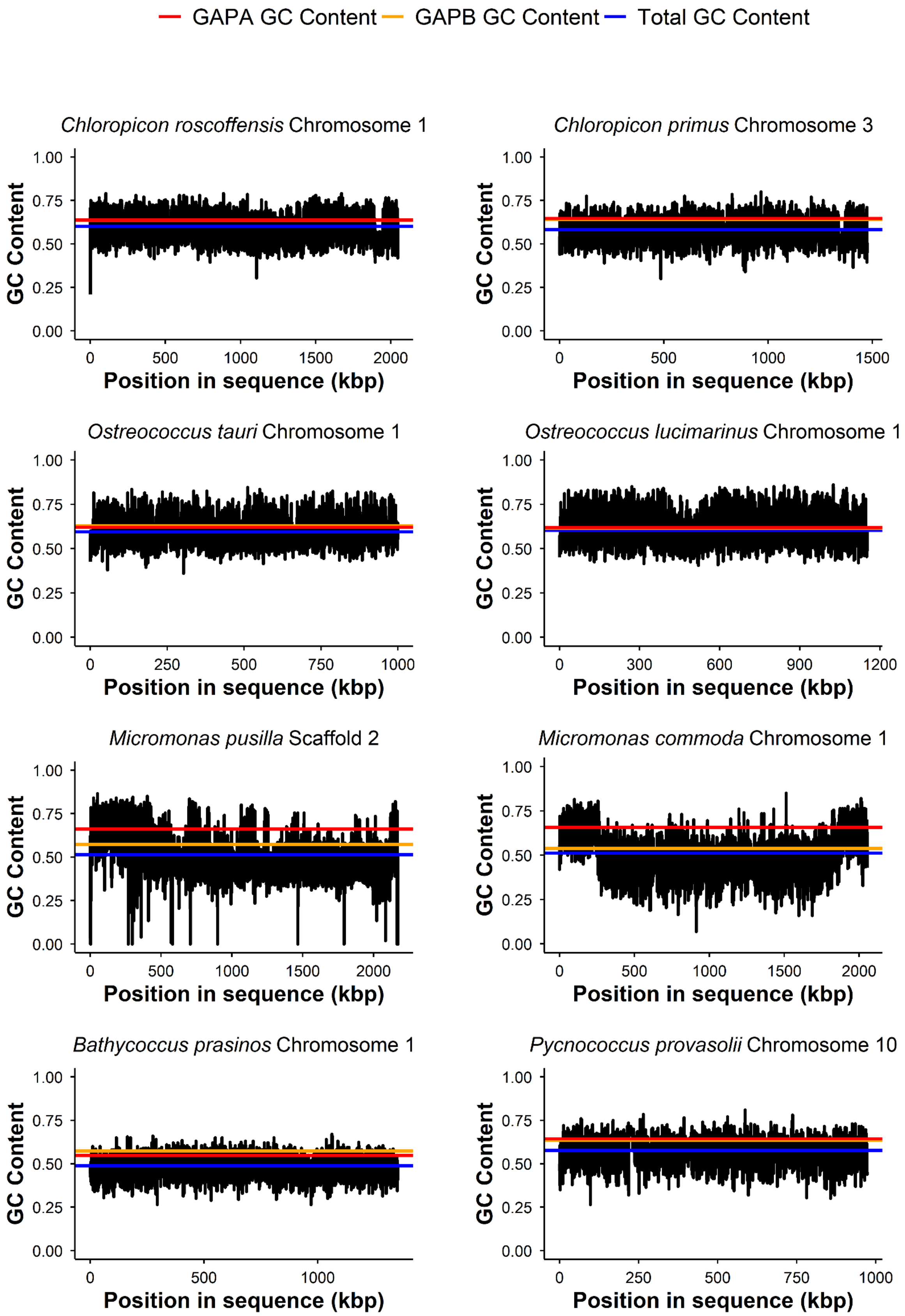


Figure S4 - Rolling GC content across Chlorophyta chromosomes/scaffolds containing GAPB, using a 200-nucleotide window (black line). GC content of the GAPA coding sequence (red), GAPB coding sequence (yellow), and average chromosomal GC content (blue) are also shown.

| | | |
|---|---|---|
| Chlorophyceae | *Chlamydomonas reinhartii* | MMLTKSVVISRPAV--------------------RPVSTRRAVVVRASGQPAVDLNKKVQDAVKEAED |
| Ulvophyceae | *Caulerpa cylindracea* | MTSSSVLSTRIPVRAAASQKGHFRIPQSFCRVQPLRLRRYPSFVLHAE--------PDVEGAIKEAEE |
| Trebouxiophyceae | *Chlorella vulgaris* | ----MQSLTVRPAVCARP---------MGARVAARPAARPSRVVMM-----SAPKKEAIEAAIKEAEE |
| Chlorodendrophyceae | *Tetraselmis sp. GSL018* | -MAAFTMTASRPALASRAA----PVTGLRARVTRRAPLRGARLAMRAE---PSKLADQVEKAISDAED |
| Pedinophyceae | *Pedinophyceae sp. YPF-701* | -MIAARVMTYKPAAVAMAQ-----------------RRQRRAVAARAN---RPEAKDQIEAAVEEAKA |
| Chloropicophyceae | *Chloropicon roscoffensis* | -MNAARVTSTRATAMRAAR----SLPSPAMRLARRAVSRRT-VSIRSK---ADELSDVLADAIDEAKE |
| Chloropicophyceae | *Chloropicon primus* | -MNAGRVSRAVGVRGVRAS--------ASARVARVTVSRRA-VLLRSK---ASDMSDALEKAIEEAKE |
| Picocystis | *Picocystis salinarum* | -*ERRTNDVKEPPTESEERKLTRTVFFLSFGLDRTRGTSFCPPITAPRRTIEQNLDGKIEDAIKEAEE |
| Pyramimonadales | *Pterosperma cristatum* | MASAITFSAVRPAAVARVAMRKPNAVAMKAVVLKQNRTRFSVVAMS-----KPDLSDDISKAIDDAKD |

| | | |
|---|---|---|
| Chlorophyceae | *Chlamydomonas reinhartii* | ACAKG-TSADCAVAWDTVEELSAAVSHKKDAVKADVTLTDPLEA-----FCKDAPD--ADECRVYED |
| Ulvophyceae | *Caulerpa cylindracea* | ACESG-SEAECATAWDTVEELSAAKSDKEKKAER----KDPLEE-----YCEEAPD--ADECRVYED |
| Trebouxiophyceae | *Chlorella vulgaris* | SCAGG-ASGECAAAWDTVEELSAAAAHAKAASSS----TDPLEDKRVNEYCEENPA--EPECRVYDE |
| Chlorodendrophyceae | *Tetraselmis sp. GSL018* | ACEGD-DQGECATAWDEVEELSAAASHQKSAAVK----VDPLDE-----FCGDNPD--ADECRIYED |
| Pedinophyceae | *Pedinophyceae sp. YPF-701* | ACSTD-NPAACAAAWDVVEELSAEASHKKSKEAAKDI-VDPIDE-----FCKDEANVSEGECRVYED |
| Chloropicophyceae | *Chloropicon roscoffensis* | ACAEDPKKADCAVAWDNVEEISAEISHKKAAKK-----TDPLEE-----FCDDNPE--ADECRVYED |
| Chloropicophyceae | *Chloropicon primus* | TCEDDPKKADCAVAWDNVEEISAEISHKKSAKK-----VDPLED-----FCDDNPE--ADECRVYED |
| Picocystis | *Picocystis salinarum* | ACEGG-VSGECAAAWDNVEELSAEASHKRSRAK-----ADPLEE-----FCESNPD--SDECRVYDD |
| Pyramimonadales | *Pterosperma cristatum* | ACEGG-TSGECAAAWDNVEELSAEASHQKAKEVK----KDPLEE-----FCEGAPD--ADECRVYED |

Figure S5 - CP12 protein sequences from representative Chlorophyta were aligned using **MUSCLE**. Sequences included in the alignment are: *Chlamydomonas reinhardtii* (A6Q0K5.2), *Caulerpa cylindracea* (GGOA01001084.1), *Chlorella vulgaris* (XM_005851141.1), *Tetraselmis sp. GSL018* (CAE9067085.1), *Pedinophyceae sp. YPF-701* (CAG9463112.1), *Chloropicon roscoffensis* (WZN61365.1), *Chloropicon primus* (QDZ17693.1), *Picocystis salinarum* (https://phytozome-next.jgi.doe.gov/ - Pico_ML_1 QYZS01_159:15000-15932), and *Pterosperma cristatum* (GKVK01039820.1). Picocystis has a stop codon indicated by a * at position 2 and no start codon. Amino acids are colour-coded according to their chemical properties: A, D, and E are shown in red (acidic); I, L, M, V, F, and C in yellow (hydrophobic); S, T, Q, W, N, and Y in green (polar); H in pale blue; K, R, and P in light blue (basic or structurally unique); and G in purple.

Table S1 - Codon Adaption Index (CAI) of GAPA and GAPB sequences using native and foreign codon usage tables. The expected CAI at 95% confidence and population is calculated as well as the average CAI of 500 random sequences. Where sequences are considered well adapted (above the eCAI) an * is shown.

| **Sequence origin** | **A/B** | **Codon usage table** | **CAI** | | **EXPECTED CAI** | **Average** |
|---|---|---|---|---|---|---|
| ***Arabidopsis thaliana*** | A | *A. thaliana* | 0.810 | * | 0.776 | 0.727 |
| | B | *A. thaliana* | 0.780 | * | 0.761 | 0.712 |
| | B | *O. lucimarinus* | 0.486 | | 0.537 | 0.476 |
| | B | *O. tauri* | 0.500 | | 0.534 | 0.474 |
| ***Chlamydomonas reinhardtii*** | A | *C. reinhardtii* | 0.834 | * | 0.782 | 0.691 |
| ***Mesostigma viride*** | A | *M. viride* | 0.770 | * | 0.578 | 0.480 |
| | B | *M. viride* | 0.724 | * | 0.660 | 0.559 |
| | B | *O. lucimarinus* | 0.710 | | 0.800 | 0.732 |
| | B | *O. tauri* | 0.745 | | 0.822 | 0.758 |
| ***Ostreococcus tauri*** | A | *O. tauri* | 0.780 | * | 0.771 | 0.704 |
| | B | *O. tauri* | 0.780 | * | 0.768 | 0.700 |
| ***Ostreococcus lucimarinus*** | A | *O, lucimarinus* | 0.720 | | 0.753 | 0.684 |
| | B | *O. lucimarinus* | 0.720 | | 0.730 | 0.655 |

Table S2 - List of key species from each family in Chlorophyta and accession numbers for their GAPA, GAPB, and CP12 sequences. No sequences were obtained for Nephroselmidophyceae. Picocystis has a CP12 sequence although it lacks a start codon. All codes relate to NCBI codes with the exception of Picocystis CP12 which can be found at https://phytozome-next.jgi.doe.gov/.

| Family | Species | GAPA | GAPB | CP12 |
|---|---|---|---|---|
| **Chlorophyceae** | *Chlamydomonas reinhardtii* | AAA86855.1 | None | A6Q0K5.2 |
| **Ulvophyceae** | *Caulerpa cylindracea* | GGOA01006792.1 | None | GGOA01001084.1 |
| **Trebouxiophyceae** | *Chlorella vulgaris* | XM_005843042.1 | None | XM_005851141.1 |
| **Chlorodendrophyceae** | *Tetraselmis sp. GSL018* | JAC69921.1 | None | CAE9067085.1 |
| **Pedinophyceae** | *Pedinophyceae sp. YPF-701* | CAG9460018.1 | None | CAG9463112.1 |
| **Chloropicophyceae** | *Chloropicon roscoffensis* | XKU08737.1 | XKU06604.1 | WZN61365.1 |
| | *Chloropicon primus* | QDZ22693.1 | QDZ19867.1 | QDZ17693.1 |
| **Picocystis** | *Picocystis salinarum* | HBIS01006593.1 | HBIS01005272.1 | Pico_ML_1 QYZS01_159:15000-15932 |
| **Pycnococcaceae** | *Pycnococcus provasolii* | GHP02544.1 | GHP05776.1 | None |
| **Nephroselmidophyceae** | | None Found | None Found | None Found |
| **Mamiellophyceae** | *Bathycoccus prasinos* | XP_007510189.1 | XP_007515295.1 | None |
| | *Micromonas commoda* | XP_002503470.1 | XP_002507103.1 | None |
| | *Micromonas pusilla* | XM_003055613.1 | XP_003056194.1 | None |
| | *Ostreococcus tauri* | ABG85189.1 | ABG85191.1 | None |
| | *Ostreococcus lucimarinus* | ABG85190.1 | ABG85192.1 | None |
| **Pyramimonadales** | *Pterosperma cristatum* | GKVK01022717.1 | GKVK01020406.1 | GKVK01039820.1 |
| **Prasinodermophyta** | *Prasinoderma coloniale* | HBHY01022593.1 | HBHY01022594.1 | No hits |

Table S3 - List of GAPDH genes used in phylogenetic and sequence analyses, including the species name and accession number from the database of origin.

| Species | Accession | Origin |
|---|---|---|
| **Bacteria GAPA** | | |
| ***Trichodesmium erythraeum IMS101*** | ZP_00326920.1 | NCBI |
| ***Crocosphaera watsonii WH 8501*** | ZP_00175043.2 | NCBI |
| ***Synechocystis sp. PCC 6803*** | BAA18633.1 | NCBI |
| ***Synechococcus elongatus PCC 7942*** | ZP_00164786.1 | NCBI |
| **Rhodophyta GAPA** | | |
| ***Chondrus crispus*** | XP_005715477.1 | NCBI |
| ***Cyanidiococcus yangmingshanensis*** | KAK4533142.1 | NCBI |
| ***Cyanidioschyzon merolae*** | XP_005534792.1 | NCBI |
| ***Cyanidium caldarium*** | KAK4537696.1 | NCBI |
| ***Galdieria partita*** | GJQ14953.1 | NCBI |
| ***Galdieria sulphuraria*** | XP_005704189.1 | NCBI |
| ***Galdieria yellowstonensis*** | KAK4528204.1 | NCBI |
| ***Gracilaria domingensis*** | KAI0557156.1 | NCBI |
| ***Gracilariopsis chorda*** | PXF47572.1 | NCBI |
| ***Neopyropia yezoensis*** | AAP32469.1 | NCBI |
| ***Porphyra umbilicalis*** | OSX77143.1 | NCBI |
| ***Porphyridium purpureum*** | KAA8495266.1 | NCBI |
| ***Pyropia vietnamensis*** | GAB0496746.1 | NCBI |
| **Chlorophyta GAPA** | | |
| ***Astrephomene gubernaculifera*** | GFR46985.1 | NCBI |
| ***Auxenochlorella protothecoides*** | XP_011399143.1 | NCBI |
| ***Bathycoccus prasinos*** | XP_007510189.1 | NCBI |
| ***Botryococcus braunii*** | ACX37507.1 | NCBI |
| ***Chlamydomonas incerta*** | ABA01125.1 | NCBI |
| ***Chlamydomonas reinhardtii*** | AAA86855.1 | NCBI |
| ***Chlamydomonas schloesseri*** | KAG2447043.1 | NCBI |
| ***Chlamydomonas sp. UWO 241*** | KAG1667978.1 | NCBI |
| ***Chlorella desiccata (nom. nud.)*** | KAG7668553.1 | NCBI |
| ***Chlorella vulgaris*** | KAI3424429.1 | NCBI |
| ***Chloropicon maureeniae*** | HBQS01018482.1 | NCBI |
| ***Chloropicon primus*** | QDZ22693.1 | NCBI |
| ***Chloropicon roscoffensis*** | XKU08737.1 | NCBI |
| ***Coccomyxa sp. Obi*** | BDA46551.1 | NCBI |
| ***Dunaliella salina*** | ACA35006.1 | NCBI |
| ***Edaphochlamys debaryana*** | KAG2487379.1 | NCBI |
| ***Gonium pectorale*** | KXZ42927.1 | NCBI |
| ***Haematococcus lacustris*** | KAJ9529197.1 | NCBI |
| ***Micromonas commoda*** | XP_002503470.1 | NCBI |
| ***Micromonas pusilla*** | XP_003055659.1 | NCBI |
| ***Monoraphidium minutum*** | KAI8469598.1 | NCBI |

| | | |
|---|---|---|
| ***Ostreococcus lucimarinus*** | ABG85190.1 | NCBI |
| ***Ostreococcus tauri*** | ABG85189.1 | NCBI |
| ***Pedinophyceae sp. YPF-701*** | CAG9460018.1 | NCBI |
| ***Picocystis salinarum*** | HBIS01006593.1 | NCBI |
| ***Picocystis sp.*** | QYZS01000231.1 | NCBI |
| ***Pleodorina starrii*** | GLC39499.1 | NCBI |
| ***Prasinoderma coloniale*** | APraco1_1\|Chr_5:438197-439192 | Phytozome |
| ***Prasinoderma singulare*** | HBHY01022594.1 | NCBI |
| ***Prasinoderma singulare*** | HBHY01022593.1 | NCBI |
| ***Pterosperma cristatum*** | GKVK01022717.1 | NCBI |
| ***Pycnococcus provasolii*** | GHP02544.1 | NCBI |
| ***Pyramimonas obovata*** | HBFA01006951.1 | NCBI |
| ***Pyramimonas parkeae*** | HBPA01042343.1 | NCBI |
| ***Rama rupestris*** | ABD37957.1 | NCBI |
| ***Raphidocelis subcapitata*** | GBF91616.1 | NCBI |
| ***Scherffelia dubia*** | ABD37955.1 | NCBI |
| ***Tetrabaena socialis*** | PNH08612.1 | NCBI |
| ***Volvox africanus*** | GIL55974.1 | NCBI |
| ***Volvox reticuliferus*** | GIM16971.1 | NCBI |
| **Streptophyta GAPA** | | |
| ***Acacia crassicarpa*** | KAK4282162.1 | NCBI |
| ***Acacia pycnantha*** | KAI9084006.1 | NCBI |
| ***Arabidopsis thaliana*** | CAA66816.1 | NCBI |
| ***Brachypodium distachyon*** | XP_014751430.1 | NCBI |
| ***Cajanus cajan*** | XP_020206749.1 | NCBI |
| ***Chara braunii*** | GBG64235.1 | NCBI |
| ***Chlorokybus atmophyticus*** | ABD37959.1 | NCBI |
| ***Glycine max*** | ABA86963.1 | NCBI |
| ***Klebsormidium nitens*** | GAQ82109.1 | NCBI |
| ***Marchantia polymorpha subsp. ruderalis*** | OAE28074.1 | NCBI |
| ***Mesostigma viride*** | ABD37961.1 | NCBI |
| ***Nicotiana tabacum*** | XP_016453026.1 | NCBI |
| ***Oryza sativa Japonica roup*** | XP_066165528.1 | NCBI |
| ***Lathyrus oleraceus*** | P12858.2 | NCBI |
| ***Sphagnum troendelagicum*** | CAK9214952.1 | NCBI |
| ***Triticum aestivum*** | XP_044324736.1 | NCBI |
| ***Vigna mungo*** | WVZ06991.1 | NCBI |
| ***Vigna unguiculata*** | XP_027902826.1 | NCBI |
| ***Zea mays*** | NP_001105414.1 | NCBI |
| **Chlorophyta GAPB** | | |
| ***Bathycoccus prasinos*** | XP_007515295.1 | NCBI |
| ***Chloroparvula cf. pacifica RCC999*** | HBPX01015675.1 | NCBI |
| ***Chloroparvula cf. pacifica RCC999*** | HBPX01015676.1 | NCBI |
| ***Chloroparvula sp. RCC696*** | HBQD01015479.1 | NCBI |
| ***Chloropicon maureeniae*** | HBQS01006992.1 | NCBI |

| | | |
|---|---|---|
| ***Chloropicon primus*** | QDZ19867.1 | NCBI |
| ***Chloropicon roscoffensis*** | XKU06604.1 | NCBI |
| ***Micromonas commoda*** | XP_002507103.1 | NCBI |
| ***Micromonas pusilla*** | XP_003056194.1 | NCBI |
| ***Ostreococcus lucimarinus*** | ABG85192.1 | NCBI |
| ***Ostreococcus tauri*** | ABG85191.1 | NCBI |
| ***Picocystis salinarum*** | HBIS01005272.1 | NCBI |
| ***Prasinoderma coloniale*** | BPraco1_1\|Chr_20:239050-362034 | Phytozome |
| ***Pterosperma cristatum*** | GKVK01020406.1 | NCBI |
| ***Pycnococcus provasolii*** | GHP05776.1 | NCBI |
| ***uncultured Pyramimonadales*** | HBZK01030214.1 | NCBI |
| **Streptophyta GAPB** | | |
| ***Arabidopsis thaliana*** | OAP18770.1 | NCBI |
| ***Artemisia annua*** | PWA94325.1 | NCBI |
| ***Brachypodium distachyon*** | XP_003558903.2 | NCBI |
| ***Camelina sativa*** | XP_010500213.1 | NCBI |
| ***Capsella rubella*** | XP_006307502.1 | NCBI |
| ***Chara vulgaris*** | CAC80378.2 | NCBI |
| ***Chlorokybus atmophyticus*** | ABD37964.1 | NCBI |
| ***Corchorus capsularis*** | OMP00299.1 | NCBI |
| ***Digitaria exilis*** | KAF8780479.1 | NCBI |
| ***Dillenia turbinata*** | KAK6939195.1 | NCBI |
| ***Diospyros kaki*** | ASL69237.1 | NCBI |
| ***Diospyros lotus*** | XP_052203735.1 | NCBI |
| ***Durio zibethinus*** | XP_022766555.1 | NCBI |
| ***Elaeis guineensis*** | XP_010918353.1 | NCBI |
| ***Klebsormidium nitens*** | GAQ83271.1 | NCBI |
| ***Marchantia polymorpha subsp. ruderalis*** | OAE25601.1 | NCBI |
| ***Mesostigma viride*** | ABD37965.1 | NCBI |
| ***Nicotiana tabacum*** | XP_016441750.1 | NCBI |
| ***Oryza sativa Japonica group*** | XP_015630808.1 | NCBI |
| ***Pea*** | P12859.2 | NCBI |
| ***Triticum aestivum*** | XP_044383864.1 | NCBI |
| ***Zea mays*** | NP_001292788.1 | NCBI |
| ***Ziziphus jujuba*** | XP_015884058.1 | NCBI |
