# Supplementary material for: Photosynthetic glyceraldehyde 3‐phosphate dehydrogenase B is present in the last common ancestor of Viridiplantae predating the Streptophyta–Chlorophyta split
Source: New Phytol. 2026 Jul 21;251(6):3072–9. doi: 10.1111/nph.71396 (PMC13491260; doi:10.1111/nph.71396)
Supplement: Supplementary file 2 [file NPH-251-3072-s002.pdf]

New Phytologist Supporting Information (Methods)

Article title: Photosynthetic Glyceraldehyde 3-phosphate Dehydrogenase B is present in the last common ancestor of Viridiplantae predating the Streptophyta-Chlorophyta split

Authors: Alex P. Mann, James E. M. Stach, Maxim V. Kapralov, Patricia E. Lopez-Calcagno

Article acceptance date: 19 May 2026

The following Supporting Information (Methods) is available for this article:

Methods S1 – Materials and methods

Sequence acquisition

For GAPB: Sequence searches were carried out using BLAST (tblastn and protein BLAST) against NCBI databases using: *Ostreococcus tauri* GAPB (ABG85191.1), *Ostreococcus tauri* GAPA (ABG85189.1), *Arabidopsis thaliana* GAPA (CAA66816.1), *Arabidopsis thaliana* GAPB (OAP18770.1). Sequences were also identified NCBI keyword function using "glyceraldehyde-3-phosphate-dehydrogenase [A/B]". BLAST searches were carried out one family group at time to ensure that the core chlorophytes did not obscure any less abundant or more poorly sequenced species. Sequences are listed in **Error! Reference source not found.**.

Similarly, for CP12, searches were carried out using BLAST (tblastn and protein BLAST) against NCBI databases using: *Chlamydomonas reinhardtii* CP12 (A6Q0K5.2) and *Arabidopsis thaliana* CP12 (O22914.1). For Picocystis, Phytozome databases were also blasted using these sequences to identify CP12 sequences. Sequences were also identified NCBI keyword function using "CP12". Again, BLAST searches were carried out one family group at time to ensure that the core chlorophytes did not obscure any less abundant or more poorly sequenced species.

Alignments and identification.

Sequences were aligned with Seaview using a MUSCLE alignment (Gouy, Guindon & Gascuel, 2009). Alignments were carried out using *Geobacillus stearothermophilus* (hereafter *Geobacillus*) GAPA (AAA22461.1) as a reference. Sequences were confirmed as a GAP protein by looking for conservation of their core domain and initially GAPBs sorted based on their presence of CTE, followed by looking for GAPB sequence specific mutations.

Tree production

All sequences alignments were curated using Gblocks (Castresana, 2000) in the Seaview platform (v5.0) (Gouy *et al.*, 2021). Gap positions were always removed; the final strict alignment included 115 taxa and retained 308 amino acid positions.

A phylogenetic tree was constructed from the Gblocks alignment using SMS (Smart Model Selection) in PhyML (http://www.atgc-montpellier.fr) (Guindon et al., 2010; Lefort et al., 2017). The branches are supported by aLRT SH-like methods (Anisimova & Gascuel, 2006). The best-fit model was LG+G+I+F with the following parameters: substitution model: LG; equilibrium frequencies: ML-optimised; proportion of invariable sites (I): 0.255 (estimated); number of substitution rate categories: 4; gamma shape parameter (G): 0.711 (estimated). The resulting phylogenetic trees were visualised and annotated in MEGA, with aesthetic modifications such as colouring and label adjustments applied for clarity.

GC composition:

The DNA sequences of the genes were obtained using blast (tblastn) with their respective protein sequences used as template. The following sequences were used: *Chloropicon roscoffensis* GAPA (XKU06604.1) and GAPB (CP145443.1:996250–997329); *Chloropicon primus* GAPA (CP031041.1:442133–443218) and GAPB (CP031036.1:c779317–778136); *Bathycoccus prasinos* GAPA (XM_007510127.1) and GAPB (XM_007515233.1); *Micromonas pusilla* GAPA (XM_003055613.1) and GAPB (XM_003056148.1); *Micromonas commoda* GAPA (XM_002503424.1) and GAPB (XM_002507057.1); *Ostreococcus tauri* GAPA (DQ649076.1) and GAPB (DQ649078.1); *Ostreococcus lucimarinus* GAPA (DQ649077.1) and GAPB (DQ649079.1); *Pycnococcus provasolii* GAPA (BNJQ01000003.1:652657–653748) and GAPB (BNJQ01000011.1:171945–173245). The following chromosomes or scaffolds, where GAPB is located, were used for host GC content quantification, with the *Ostreococcus tauri* and *Micromonas pusilla* scaffolds serving as the host DNA: *Bathycoccus prasinos* Chromosome_1 (NC_024008.1); *Chloropicon primus* chromosome 3 (CP031036.1); *Chloropicon roscoffensis* chromosome 1 (CP145441.1); *Micromonas commoda* chromosome 1 (NC_013038.1); *Micromonas pusilla* scaffold 2 (NW_003315883.1); *Ostreococcus lucimarinus* chromosome 1 (NC_009355.1); *Ostreococcus tauri* 1 (Ostta4221_3); *Pycnococcus provasolii* chromosome 10 (OW568870.1). GC content was calculated as number of GC/total nucleotide content. A moving average was calculated for the chromosomes/scaffolds using 200 nt as a window as well as a total average.

CAI index

The Codon Adaptation Index (CAI) was used to quantify the degree of codon usage adaptation for a set of nuclear genes across several green algal and plant species. CAI values were calculated using the online CAIcal tool (http://genomes.urv.es/CAIcal/) (Puigbò, Bravo & Garcia-Vallve, 2008), which compares the codon usage of a gene against a reference set to assess potential translational efficiency. The following gene sequences were analysed: *Arabidopsis thaliana* GAPA (AY142053.1); *Arabidopsis thaliana* GAPB (BT002267.1); *Chlamydomonas reinhardtii* GAPA (XM_001689819.2); *Mesostigma viride* GAPA (DQ270265.1); *Mesostigma viride* GAPB (DQ270269.1); *Ostreococcus tauri* GAPA (DQ649076.1); *Ostreococcus tauri* GAPB (DQ649078.1); *Ostreococcus lucimarinus* GAPA (DQ649077.1) and *Ostreococcus lucimarinus* GAPB (DQ649079.1). CAI values were computed individually for each gene using codon usage tables sourced from

the Kazusa Codon Usage Database (https://www.kazusa.or.jp/codon/), accessed using the following species codes: *Arabidopsis thaliana* (3702); *Chlamydomonas reinhardtii* (3055); *Mesostigma viride* (1882); *Ostreococcus tauri* (0448) and O*streococcus lucimarinus* (6017). Each gene was analysed against the codon usage table of its corresponding species to evaluate adaptation within the same organism. Additionally, *Mesostigma viride* and *Arabidopsis* genes were evaluated against *Ostreococcus* reference tables. Standard genetic code, Markov method, a 95% confidence and 95% population were used in all cases.

References:

Anisimova M, Gascuel O. 2006. Approximate Likelihood-Ratio Test for Branches: A Fast, Accurate, and Powerful Alternative. Systematic Biology **55**(4): 539-552.

Castresana J. 2000. Selection of Conserved Blocks from Multiple Alignments for Their Use in Phylogenetic Analysis. Molecular Biology and Evolution **17**(4): 540-552.

Gouy M, Guindon S, Gascuel O. 2009. SeaView Version 4: A Multiplatform Graphical User Interface for Sequence Alignment and Phylogenetic Tree Building. Molecular Biology and Evolution **27**(2): 221-224.

Gouy M, Tannier E, Comte N, Parsons DP. 2021. Seaview Version 5: A Multiplatform Software for Multiple Sequence Alignment, Molecular Phylogenetic Analyses, and Tree Reconciliation. Methods Mol Biol **2231**: 241-260.

Guindon S, Dufayard JF, Lefort V, Anisimova M, Hordijk W, Gascuel O. 2010. New algorithms and methods to estimate maximum-likelihood phylogenies: assessing the performance of PhyML 3.0. Syst Biol. 593:307–321.

Lefort V., Longueville J.E., Gascuel O. 2017. SMS: Smart Model Selection in PhyML, Molecular Biology and Evolution, 34 (9):2422–2424.

Puigbò P, Bravo IG, Garcia-Vallve S. 2008. CAIcal: a combined set of tools to assess codon usage adaptation. *Biology Direct* 3: 38.
